# Role of Mitochondrial Cytochrome P450 2E1 in Healthy and Diseased Liver

**DOI:** 10.3390/cells11020288

**Published:** 2022-01-15

**Authors:** Julie Massart, Karima Begriche, Jessica H. Hartman, Bernard Fromenty

**Affiliations:** 1 INSERM, Univ Rennes, INRAE, Institut NUMECAN (Nutrition Metabolisms and Cancer) UMR_A 1341, UMR_S 1241, F-35000 Rennes, France; julie.massart@inserm.fr (J.M.); karima.begriche@univ-rennes1.fr (K.B.); 2 Department of Biochemistry and Molecular Biology, Medical University of South Carolina, Charleston, SC 29425, USA; hartmanj@musc.edu

**Keywords:** mitochondria, cytochrome P450 2E1, alcohol-associated liver disease, drug-induced liver injury, nonalcoholic fatty liver disease

## Abstract

Cytochrome P450 2E1 (CYP2E1) is pivotal in hepatotoxicity induced by alcohol abuse and different xenobiotics. In this setting, CYP2E1 generates reactive metabolites inducing oxidative stress, mitochondrial dysfunction and cell death. In addition, this enzyme appears to play a role in the progression of obesity-related fatty liver to nonalcoholic steatohepatitis. Indeed, increased CYP2E1 activity in nonalcoholic fatty liver disease (NAFLD) is deemed to induce reactive oxygen species overproduction, which in turn triggers oxidative stress, necroinflammation and fibrosis. In 1997, Avadhani’s group reported for the first time the presence of CYP2E1 in rat liver mitochondria, and subsequent investigations by other groups confirmed that mitochondrial CYP2E1 (mtCYP2E1) could be found in different experimental models. In this review, we first recall the main features of CYP2E1 including its role in the biotransformation of endogenous and exogenous molecules, the regulation of its expression and activity and its involvement in different liver diseases. Then, we present the current knowledge on the physiological role of mtCYP2E1, its contribution to xenobiotic biotransformation as well as the mechanism and regulation of CYP2E1 targeting to mitochondria. Finally, we discuss experimental investigations suggesting that mtCYP2E1 could have a role in alcohol-associated liver disease, xenobiotic-induced hepatotoxicity and NAFLD.

## 1. Introduction

Cytochromes P450 (CYPs) are phase I enzymes involved in the metabolism of numerous endogenous and exogenous compounds. These enzymes are mainly expressed in the liver but are also present in other tissues including gut, kidney, lung, nasal mucosa, peripheral blood mononuclear cells (PBMCs), male and female reproductive tissues, adrenal glands and brain. Among the different CYPs expressed in humans, hepatic cytochrome P450 2E1 (CYP2E1, formerly referred to as P450j) has a unique place in liver pathophysiology. Indeed, CYP2E1 is central to the occurrence of different acute and chronic liver diseases induced by drugs, toxicants and alcohol abuse [1,2]. Moreover, there is increasing evidence that CYP2E1 plays a significant role in obesity-associated nonalcoholic fatty liver disease (NAFLD), in particular by favoring the progression of simple fatty liver (i.e., steatosis) to nonalcoholic steatohepatitis (NASH) [2,3]. Although the historically more studied localization of CYP2E1 is the endoplasmic reticulum (ER), this enzyme is also found in significant amounts in mitochondria [4,5]. This location is not trivial because mitochondria are the powerhouses of the cells and as such, CYP2E1-induced impairment of mitochondrial function can have dire consequences on cell homeostasis. In this review, we first recall several critical features of CYP2E1, including regulation of its expression, its role in the biotransformation of endogenous and exogenous molecules and its involvement in different liver diseases. Then, we discuss in depth the current available data on mitochondrial CYP2E1 (mtCYP2E1), in particular regarding xenobiotic biotransformation and its emerging role in different liver diseases.

## 2. General Features of CYP2E1 

In this section, we review important features of CYP2E1 but readers who want to get further information on this enzyme are invited to peruse several excellent reviews [1,2,6,7,8,9]. Beforehand, it should be underscored that a significant part of the knowledge gathered so far on CYP2E1 was actually inferred from studies that did not discriminate between CYP2E1 located in the endoplasmic reticulum (ER, also referred to as microsomal CYP2E1) and mtCYP2E1. Indeed, most investigations carried out in standard cultured hepatic cells, liver homogenates, whole animals and patients provided global information on CYP2E1 regardless of its intracellular localization. However, because ER is the best-known localization of CYP2E1, it is tempting to speculate that what was reported on this enzyme in most articles actually reflects the microsomal localization. The specific features of mtCYP2E1 will be provided later on in the review.

### 2.1. Biotransformation of Endogenous and Exogenous Molecules

CYP2E1 is an enzyme whose activity in the ER is coupled to the activity of flavoprotein NADPH-cytochrome P450 reductase, which transfers the NADPH electrons to CYP2E1 during its catalytic cycle and to cytochrome b5, which stimulates its activity [1]. However, the electron donor system is different for mtCYP2E1, as discussed later on. Nonetheless, CYP2E1 activity requires molecular oxygen regardless of its localization.

Hepatic CYP2E1 catalyzes the biotransformation of both endogenous molecules and exogenous compounds (hereafter referred to as xenobiotics). Regarding endogenous molecules, CYP2E1 can metabolize acetone [6], which is one of the three ketone bodies generated via the mitochondrial β-oxidation of fatty acids. CYP2E1 also metabolizes glycerol and different fatty acids including saturated C12 to C18 fatty acids and some polyunsaturated fatty acids such as arachidonic acid and epoxyeicosatrienoic acids [6,10]. Interestingly, recent investigations uncovered that CYP2E1 could play a significant role in adipocyte lipid metabolism [11], thus suggesting that CYP2E1 involvement in lipid metabolism takes place in tissues able to accumulate lipids in normal and pathologic conditions. CYP2E1 also oxidizes ethanol to acetaldehyde [2,9]. Although ethanol can be consumed via diverse alcoholic beverages, this 2-carbon alcohol is also produced by the gut microbiota, sometimes in amounts high enough to cause liver injury [12].

CYP2E1 also metabolizes numerous xenobiotics including drugs and environmental toxicants. Examples of drugs are the pain killer acetaminophen (paracetamol or APAP), the antibiotic isoniazid, the volatile anesthetics halothane, enflurane and isoflurane [1,6,9], the nonsteroidal anti-inflammatory drug salicylic acid [13] and the antiepileptic drug valproic acid [14]. Examples of environmental toxicants include carbon tetrachloride (CCl_4_), vinyl chloride, styrene, thioacetamide and different nitrosamines [1,6,9]. The endocrine disruptor bisphenol A can also be metabolized by CYP2E1 [15,16]. Of note, CYP2E1-mediated biotransformation of most of the aforementioned xenobiotics generates toxic metabolites [1,6,9,16].

In addition to the formation of toxic metabolites from xenobiotics, CYP2E1 also generates superoxide anion and hydrogen peroxide from oxygen during its catalytic cycle [2,6]. These CYP2E1-generated reactive oxygen species (ROS) appear to play a major role in the pathophysiology of alcohol-associated liver disease (AALD) and NAFLD, in particular by triggering lipid peroxidation and mitochondrial dysfunction [2,17,18].

### 2.2. Regulation of CYP2E1 Expression and Activity

Physiological expression of hepatic CYP2E1 is mainly regulated by the transcription factors hepatocyte nuclear factor 1-α (HNF–1α) [19,20] and β-catenin [21], although recent investigations uncovered a significant role of Krüppel-Like Factor 15 (KLF15) [22]. In addition, other transcription factors regulate hepatic CYP2E1 expression in pathophysiological conditions such as nuclear factor–κB (NF–κB) during inflammatory states [23] and tonicity-responsive enhancer binding protein (TonEBP) under hyperosmotic stress [24]. Hepatic *CYP2E1* mRNA levels are also post-transcriptionally regulated through protein interaction [25] and by microRNAs [2]. It should be noted that in many cases, changes in *CYP2E1* mRNA levels do not reflect changes in protein and activity.

This discordance between mRNA and protein/activity is due to the fact that CYP2E1 expression can also be regulated at the translational and post-translational levels, as extensively discussed in previous reviews [26,27]. Post-translational regulation of CYP2E1 includes phosphorylation [26,28], ubiquitination [26,29] and sumoylation [30]. Interestingly, CYP2E1 phosphorylation seems to modulate its catalytic activity [28] but also its ubiquitin-dependent proteasomal degradation [29]. Finally, it is worth mentioning that many CYP2E1 substrates including ethanol and glycerol protect the enzyme against its degradation, possibly via reduced ubiquitin-mediated proteolysis [27,31].

Different molecules modulate CYP2E1 expression and activity in hepatocytes such as fatty acids and ketone bodies (acetone, β–hydroxybutyrate and acetoacetate) [3,32], ethanol, hormones (e.g., insulin, glucagon and leptin) [26,27] and cytokines (e.g., interleukin-4 and tumor necrosis factor–α) [33]. Unfortunately, except for some molecules (e.g., acetone, ethanol and interleukin–4) [26,27,33], the exact mechanisms underlying the regulation of CYP2E1 expression and activity remain to be determined.

### 2.3. Role of CYP2E1 in Liver Diseases

CYP2E1 plays a key role in the hepatotoxicity induced by different drugs such as APAP, cisplatin, halothane and other halogenated anesthetic agents [7,9]. For instance, CYP2E1-mediated biotransformation of APAP generates *N*–acetyl–*p*–benzoquinone imine (NAPQI), a highly reactive metabolite inducing major oxidative stress, mitochondrial dysfunction and cell death, especially in the context of APAP overdose [7,34]. Importantly, the covalent binding of NAPQI to mitochondrial proteins is deemed to play a major role in APAP-induced mitochondrial dysfunction and oxidative stress [34]. CYP2E1 also plays an important role in liver injury caused by thioacetamide, vinyl chloride and carbon tetrachloride [9,35]. CYP2E1 is pivotal in the pathophysiology of AALD, in particular via the generation of acetaldehyde, ROS and different free radicals such as 1–hydroxyethyl and hydroxyl radicals, as discussed in several recent reviews [2,8,9,36]. Lastly, CYP2E1 seems to favor the progression of obesity-associated fatty liver to NASH, which is characterized by necroinflammation and fibrosis [2,3,37,38]. The mechanism whereby CYP2E1 favors NAFLD progression is currently unknown although it is conceivable that CYP2E1-mediated ROS production could play a significant role [3,37]. Of note, CYP2E1 can produce ROS from oxygen even in the absence of substrates, via a futile cycle [2,39]. In NAFLD, CYP2E1 might also be involved in the production of toxic lipid intermediates such as dicarboxylic acids [3,17].

Interestingly, CYP2E1 expression and activity are enhanced in both AALD and NAFLD. In AALD, induction of CYP2E1 is thought to be mainly due to an inhibition of its degradation by ethanol, as previously mentioned [27,31]. On the contrary, the mechanism of CYP2E1 induction in NAFLD is currently unknown. It has been hypothesized that some fatty acids, hyperleptinemia and insulin resistance might be involved [2,3,32] but the downstream signaling pathways leading to CYP2E1 induction have not been characterized yet. CYP2E1 induction in NAFLD might be an adaptive mechanism in order to limit lipid accumulation since CYP2E1 can metabolize different fatty acids via (ω–1)–hydroxylation and to a lesser extent via ω–hydroxylation [10,40]. However, some hydroxylated fatty acids generated by CYP2E1 are then transformed into cytotoxic dicarboxylic acids [3].

## 3. mtCYP2E1 in Healthy Liver

### 3.1. Discovery and General Features of mtCYP2E1

In 1997, Avadhani’s group at the University of Pennsylvania discovered for the first time the presence of CYP2E1 in rat liver mitochondria, mostly located in the vicinity of the mitochondrial inner membrane [41]. Later on, mtCYP2E1 was purified from rat liver mitochondria and further investigations allowed to identify a full-length ~52 kDa protein with the same catalytic activity as microsomal CYP2E1, as assessed with *p*-nitrophenol and dimethylnitrosamine, two prototypical CYP2E1 substrates [42]. However, mtCYP2E1 activity was found to be supported exclusively by mitochondrial adrenodoxin reductase and adrenodoxin instead of NADPH-cytochrome P450 reductase [42]. Of note, mtCYP2E1 in the presence of adrenodoxin reductase and adrenodoxin yielded the same range of activity as microsomal CYP2E1 in the presence of NADPH-cytochrome P450 reductase [42], thus indicating that adrenodoxin reductase has an efficient capacity of electron transfer towards mtCYP2E1. In rat and mouse liver, mtCYP2E1 expression and/or activity represents 10% to 40% of microsomal CYP2E1 and the mitochondrial protein is characterized by a higher level of phosphorylation compared to its microsomal counterpart [5,42,43]. Importantly, the presence of CYP2E1 in liver mitochondria was confirmed by other teams [5,44,45]. It is also worthy to mention that mtCYP2E1 is also found in the brain [5] and in adipocytes [11], suggesting that it might also have a biological function in these extra-hepatic tissues.

### 3.2. Physiological Role of mtCYP2E1

Although the physiological role of mtCYP2E1 is still unknown, some hypotheses can be put forward. First, an important role in hepatic gluconeogenesis is conceivable since CYP2E1 metabolizes the ketone body acetone to acetol, a precursor of glucose in the gluconeogenic pathway [46]. Second, mtCYP2E1 might aid the mitochondrial β–oxidation pathway in a situation of lipid overload. Third, mtCYP2E1 might generate signaling molecules from ketone bodies. Indeed, recent investigations suggested that CYP2E1-mediated oxidation of the ketone body acetoacetate produced acetate, which exerts paracrine metabolic effects via the free fatty acid receptor 2 (FFAR2, also known as GPR43) [47]. However, the validation of these hypotheses first requires the demonstration that mtCYP2E1 can metabolize fatty acids and ketone bodies as efficiently as microsomal CYP2E1.

### 3.3. Role of mtCYP2E1 in Xenobiotic Biotransformation and ROS Generation

Contrasting to its unknown function in liver physiology, there is now strong evidence that mtCYP2E1 can metabolize different xenobiotics. For instance, mtCYP2E1 was shown to catalyze the N-demethylation of dimethylnitrosamine and the hydroxylation of *p*-nitrophenol, as previously mentioned [42]. In addition, mtCYP2E1 can metabolize aniline and styrene to 4-amino-phenol and styrene oxide, respectively [48]. Interestingly, the kinetic profiles of aniline and styrene biotransformation are different between mtCYP2E1 and microsomal CYP2E1, suggesting that the subcellular localization of CYP2E1 might yield functionally distinct enzymes [48]. Another major difference is that mtCYP2E1 activity is not coupled to phase II enzymes such as UDP-glucuronosyltransferases (UGTs) and sulfotransferases, contrary to what happens in the ER. For instance, CYP2E1-mediated chlorzoxazone hydroxylation is tightly coupled to the formation of chlorzoxazone-O-glucuronide catalyzed by different UGTs, which are located in the ER [49]. In mitochondria, this lack of coupling between CYP2E1 and phase II enzymes could impair the detoxification pathways of some CYP2E1-generated metabolites. This might occur for instance with APAP, as discussed later on.

Another important consideration for xenobiotic metabolism by mtCYP2E1 is the ability of different substrates and their oxidative products to cross the mitochondrial double membrane. Most CYP2E1 substrates are low molecular weight and hydrophobic and thus expected to easily diffuse across the membranes. However, certain substrates and the more polar oxidized metabolites from CYP2E1 metabolism are less prone or unable to cross membranes. Thus, the structure of the substrate must be considered in predicting metabolism by mtCYP2E1. Furthermore, oxidized metabolites may be more likely to be retained in mitochondria and, if they are reactive metabolites, preferentially modify mitochondrial macromolecules.

Besides xenobiotic metabolism, mtCYP2E1 appears to generate significant amounts of ROS [50]. While an excessive production of mitochondrial ROS can be deleterious in different chronic liver diseases by promoting oxidative stress and lipid peroxidation, as discussed below, it should be mentioned that acute ROS overproduction may serve as signaling molecules between mitochondria and other cell compartments [18]. Hence, mtCYP2E1-mediated ROS production might have beneficial actions in some physiological circumstances.

### 3.4. Mechanisms of CYP2E1 Targeting to Mitochondria and Its Physiological Regulation

Two reviews previously described in depth the mechanisms of CYP2E1 targeting to mitochondria [5,50]. Hence, only the main features of this process are discussed in this section. Briefly, CYP2E1 undergoes a protein kinase A (PKA)-dependent phosphorylation on Ser129, which results in the activation of a cryptic mitochondrial targeting signal composed of two positively charged amino acids, Lys24 and Lys25 [4]. This in turn promotes in the cytoplasm the association of CYP2E1 with the heat shock proteins HSP70 and HSP90. Next, the resulting complex interacts with the translocase of outer mitochondrial membrane 70 (TOM70) and then binds to TOM40. Finally, CYP2E1 interacts with the translocase of the inner mitochondrial membrane 23 (TIM23) complex and the protein is finally pulled inside the matrix thanks to mitochondrial HSP70 [4,5,50].

Although the physiological regulation of CYP2E1 targeting to mitochondria is currently poorly understood, previous investigations identified some factors controlling this process [5,51]. As previously mentioned, CYP2E1 targeting to mitochondria is controlled by PKA, which is activated by cyclic AMP (cAMP) [4,5]. Hence, glucagon might increase mtCYP2E1 in the liver since this hyperglycemic hormone activates PKA. In keeping with this hypothesis, hepatic mtCYP2E1 is increased in streptozotocin-induced diabetic rats [52], a rodent model of type 1 diabetes characterized by hyperglucagonemia [53]. Likewise, fasting might also enhance hepatic mtCYP2E1 since this metabolic state is associated with increased glucagonemia [54]. In addition to glucagon, the satiety hormone leptin might also control mtCYP2E1 localization, possibly via a PKA-dependent mechanism [55]. Fatty acids and ketone bodies might also regulate mtCYP2E1 levels, as previously proposed [51]. The role of leptin, fatty acids and ketone bodies will be further discussed later on. Finally, genetic polymorphism in the N-terminal targeting sequence of CYP2E1 was reported to greatly affect the mitochondrial localization of the enzyme [56], although the prevalence of such N-terminal polymorphic variants is still unknown. In the subset of 15 livers included in the study, the hepatic content of mtCYP2E1 appeared to be lower than microsomal CYP2E1 in most people, but the converse was observed in five individuals [56]. A larger study including more individuals is needed to properly characterize this variability.

## 4. mtCYP2E1 in Liver Diseases

### 4.1. Ethanol Toxicity and AALD

AALD encompasses a large spectrum of liver lesions including microvesicular and macrovacuolar steatosis, steatohepatitis, extensive fibrosis, cirrhosis and hepatocellular carcinoma (HCC) [57,58]. AALD is the primary cause of liver-related mortality and the leading indication for liver transplantation [58]. As already mentioned, CYP2E1 plays a major role in the development and progression of AALD, in particular via the generation of acetaldehyde, free radicals and ROS [2,8,9,36]. Importantly, ROS can trigger peroxidation of polyunsaturated fatty acids, thus leading to the generation of highly cytotoxic aldehydes such as malondialdehyde and 4-hydroxynonenal (4-HNE) [59]. ROS can oxidatively damage other cellular components including mitochondrial DNA [60] and activate different stress-related kinases such as the c-Jun NH2-terminal kinase (JNK) and p38 mitogen-activated protein kinase (MAPK) [61]. Hence, regardless of their sources, ROS overproduction can eventually induce severe mitochondrial dysfunction and cell death [61,62].

Besides CYP2E1, the cytosolic enzyme alcohol dehydrogenase (ADH) can metabolize ethanol to acetaldehyde in the liver. Notably, hepatic ADH has a much lower Km for ethanol than CYP2E1 (0.2–2 mM vs. 8–10 mM), and thus ADH accounts for a large part of total ethanol metabolism in physiological conditions [8]. Nevertheless, the role of CYP2E1 in ethanol metabolism is much greater in AALD due to increased levels of this CYP after chronic alcohol consumption [8,17]. Acetaldehyde generated by ADH and microsomal CYP2E1 enters mitochondria and is subsequently metabolized by aldehyde dehydrogenase (ALDH) to acetate, which can be oxidized by the tricarboxylic acid cycle for energy production [8]. Of note, the presence of CYP2E1 in mitochondria induces a local production of acetaldehyde, which is added to the aldehyde pool coming from the cytosol. Whereas acetaldehyde is almost fully oxidized in physiological conditions, excessive alcohol consumption significantly increases the cellular levels of this aldehyde, which plays a significant role in AALD via the generation of protein and DNA adducts [9,17].

To the best of our knowledge, ethanol-induced increased mtCYP2E1 levels in mouse liver and primary rat hepatocytes were first reported in 2005 [55]. Of note, some in vivo investigations were carried out in lean and obese leptin-deficient ob/ob mice intoxicated with ethanol by gavage (2.5 g/kg daily) for 4 consecutive days. In lean mice, this short protocol of alcohol intoxication enhanced CYP2E1 activity in liver microsomes and mitochondria and this was associated with lower levels of reduced glutathione (GSH) in both cytosol and mitochondria. In contrast, alcohol intoxication in leptin-deficient obese mice increased CYP2E1 activity only in liver microsomes but not in mitochondria, thus suggesting that leptin deficiency and/or obesity might hamper mitochondrial targeting of induced CYP2E1. Interestingly, this was associated with decreased GSH levels in cytosol but not in mitochondria. Altogether, these findings in mice strongly suggested that ethanol-induced increased mtCYP2E1 expression in the liver could induce significant oxidative stress in mitochondria [55]. Investigations in cultured rat hepatocytes incubated with high ethanol concentration (100 mM) also demonstrated that mtCYP2E1 levels were much more stable than microsomal levels, which rapidly declined over the 72-h period of culture. This greater stability led us to propose that mtCYP2E1 might be protected from proteasomal degradation [55].

Subsequent investigations in other rodent models of alcohol intoxication confirmed increased mtCYP2E1 levels in the liver [44,63,64,65]. Interestingly, some of these investigations showed that mitochondrial levels of the lipid peroxidation product 4-HNE paralleled mtCYP2E1 expression [63], reinforcing the proposal that higher mtCYP2E1 expression could induce oxidative stress in mitochondria [55]. However, it should be mentioned that previous investigations in PC12 cells showed that 4–HNE enhanced mtCYP2E1 levels and activity in a concentration-dependent manner [66]. Hence, one cannot exclude the possibility that mtCYP2E1 does not cause lipid peroxidation in mitochondria but rather that 4-HNE generated outside the mitochondrial compartment subsequently increases mtCYP2E1 levels. These hypotheses are however not mutually exclusive.

In most aforementioned investigations, ethanol-induced increased mtCYP2E1 content was associated with higher microsomal CYP2E1 levels [44,55,63,64]. Hepatic *CYP2E1* mRNA levels were significantly increased in one of these studies [63], a finding in line with some other investigations showing that ethanol can enhance *CYP2E1* mRNA levels in rodent liver [67,68] and in cultured HepaRG cells [69]. Ethanol could thus enhance CYP2E1 protein levels via increased *CYP2E1* gene transcription [70] and reduced ubiquitin-mediated proteolysis, as previously mentioned [27,31]. Thus, the question arises as to whether ethanol-induced increased mtCYP2E1 is just the mere stochiometric consequence of higher *CYP2E1* expression in hepatocytes, or specifically results from enhanced CYP2E1 targeting to mitochondria. It also cannot be excluded that both mechanisms coexist. Unfortunately, no investigations have been carried out in order to determine which mechanism prevails. Further studies will be needed to address this question, in particular to determine whether ethanol intoxication increases CYP2E1 phosphorylation on Ser129. Indeed, previous investigations reported that ethanol increased cAMP levels in some experimental conditions [71], which might favor PKA-mediated CYP2E1 phosphorylation on Ser129 and its targeting to mitochondria.

In addition to the aforementioned investigations reporting ethanol-induced increase in mtCYP2E1, other studies explored ethanol-induced toxicity in COS-7 cells overexpressing mtCYP2E1 [64,72]. In a first study, ethanol-induced toxicity in COS-7 cells overexpressing CYP2E1 in mitochondria (Mt^+^ cells) was compared with wild-type cells and cells overexpressing CYP2E1 specifically in the ER (ER^+^ cells) [64]. Interestingly, 100 mM ethanol significantly increased ROS production and reduced cellular and mitochondrial GSH levels in Mt^+^ cells whereas no significant effects were observed in wild-type and ER^+^ cells [64]. In a second study, the detrimental effects of different concentrations of ethanol were investigated in COS-7 cells expressing CYP2E1 in mitochondria only (Mito/CYP2E1 cells) or in both ER and mitochondria [72]. This study showed that the exclusive localization of CYP2E1 within mitochondria in the Mito/CYP2E1 cells was sufficient to induce ROS overproduction, depletion of GSH, mitochondrial dysfunction and cytotoxicity [72]. Notably, these ethanol-induced deleterious effects in Mito/CYP2E1 cells occurred despite lower cellular level and activity of CYP2E1 when compared to cells expressing CYP2E1 in both ER and mitochondria [72].

**Figure 1:**
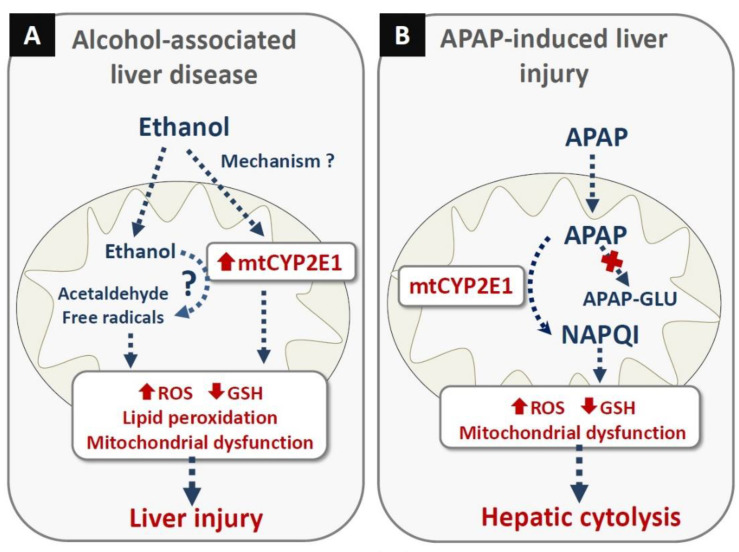
Possible role of mitochondrial CYP2E1 in hepatotoxicity induced by ethanol abuse and acetaminophen intoxication. (**A**) In alcohol-associated liver disease, mitochondrial CYP2E1 (mtCYP2E1) could be involved by two different mechanisms. First, ethanol intoxication increases mtCYP2E1 levels, which can induce reactive oxygen species (ROS) overproduction, Second, mtCYP2E1 might contribute to the generation of acetaldehyde and free radicals such as the 1-hydroxyethyl and hydroxyl radicals. ROS, and possibly acetaldehyde and free radicals, cause reduced glutathione (GSH) depletion, lipid peroxidation and mitochondrial dysfunction. Altogether, these deleterious events contribute to ethanol-induced liver injury. (**B**) In acetaminophen (APAP) intoxication, mtCYP2E1 generates *N*-acetyl-*p*-benzoquinone imine (NAPQI), a highly reactive metabolite inducing ROS overproduction, depletion of GSH and major mitochondrial dysfunction, which causes massive hepatic cytolysis. The lack of UDP-glucuronosyltransferases in mitochondria does not allow the generation of APAP-glucuronide (APAP-GLU), which is a non-toxic APAP metabolite. Importantly, the involvement of mtCYP2E1 in alcohol and APAP-induced hepatotoxicity does not exclude the role of extramitochondrial CYP2E1 in ROS overproduction and reactive intermediate generation.

Overall, these in vivo and in vitro findings strongly support the view that mtCYP2E1 plays a major role in ethanol-induced toxicity and AALD by generating ROS and toxic lipid peroxidation products (Figure 1A). Nevertheless, it will be important to determine whether mtCYP2E1 can generate acetaldehyde and free radicals, which might also contribute to liver injury (Figure 1A). It will also be crucial to distinguish the relative contributions of mtCYP2E1 and the mitochondrial electron transport chain to ROS production.

### 4.2. Liver Injury Induced by Drugs and Other Xenobiotics

In contrast to ethanol toxicity, there is much fewer information regarding the role of mtCYP2E1 in liver injury induced by drugs and other xenobiotics.

To the best of our knowledge, APAP is the only drug for which there are some available data on this matter. Indeed, the investigations carried out in Mito/CYP2E1 COS-7 cells (vide supra) treated with APAP showed that the exclusive localization of CYP2E1 within mitochondria was sufficient to induce ROS overproduction, depletion of GSH, mitochondrial dysfunction and cytotoxicity (Figure 1B) [72]. However, investigations in rats and mice treated with APAP showed that levels of mitochondrial APAP-protein adducts in the liver were apparently not correlated with the basal content of hepatic mtCYP2E1 [73], suggesting that microsomal CYP2E1 might also be involved in the formation of these deleterious adducts. Alternatively, mtCYP2E1 protein levels in rats and mice might not always be correlated with mtCYP2E1 activity. Possible explanations for the discrepancy could be the presence of endogenous or exogenous inhibitors of mtCYP2E1 and/or the availability of NADH/NADPH. Of note, the lack of UGT expression in mitochondria might favor mtCYP2E1-mediated generation of NAPQI (Figure 1B). Hence, further investigations are needed to determine to which extent mtCYP2E1 contributes to the formation of mitochondrial APAP-protein adducts and subsequent mitochondrial dysfunction, oxidative stress and liver injury. Finally, other CYPs in mitochondria might be involved in the formation of mitochondrial APAP-protein adducts. For instance, mitochondria contain CYP1A2 [41], which is also involved in NAPQI generation from APAP [34].

Likewise, only scarce data are available on the contribution of mtCYP2E1 in the hepatotoxicity of other xenobiotics. A recent study carried out in a large panel of inbred mouse strains exposed to the environmental toxicant 1,3-butadiene reported that hepatic mtCYP2E1 activity was significantly correlated with reduced activity of the mitochondrial respiratory complex I, II and IV [43]. Interestingly, this correlation was only observed post-exposure and was not observed for microsomal CYP2E1 activity [43]. Unfortunately, exploratory proteomic experiments did not reveal the presence of mitochondrial proteins covalently modified by 1,3-butadiene epoxide metabolites [43]. It should be noted that in the study, adducted mitochondrial proteins were not enriched from the mitochondrial pool, and therefore would be very difficult or impossible to detect the modifications. Further experiments would be needed to determine whether the lack of detection was due to experimental design, or could reflect a real lack of modification. One consideration for metabolites such as the epoxide metabolites for 1,3-butadiene is the presence of mitochondrial epoxide hydrolases, which might have detoxified the epoxide metabolites. It is possible that mitochondrial epoxide hydrolase enzymes may have differing specificity for substrates compared to those in other subcellular compartments, which may contribute to differential toxicity between organelles. Lastly, this study did not determine whether 1,3-butadiene-induced mitochondrial toxicity was associated with significant liver injury.

Other investigations in rodents treated with the prototypical CYP2E1 inducer pyrazole showed that mtCYP2E1 induction was much higher compared to microsomal CYP2E1 [42,74]. mtCYP2E1 induction might generate high levels of mitochondrial ROS able to oxidatively inactivate the antioxidant enzyme peroxiredoxin III [74]. Whether pyrazole-induced increased mtCYP2E1 levels participate to liver injury has not been addressed yet. It is worth mentioning that many xenobiotics including drugs contain a pyrazole ring. Thus, further investigations will be required to determine if some of these xenobiotics also induce hepatic mtCYP2E1.

Lastly, mtCYP2E1 might be involved in liver toxicity induced by coumarin, a natural compound present in many plants and found at high concentrations in Tonka beans. Because of its pleasant odor, coumarin is used as an enhancing agent in perfumes and is added to toilet soap and detergents, toothpastes and some alcoholic beverages [75]. Investigations in rats showed that a single dose of coumarin increased mitochondrial dysfunction and *CYP2E1* expression [76]. However, repeated administration of coumarin for 4 consecutive days reduces significantly coumarin-induced mitochondrial dysfunction and hepatocellular necrosis observed with a single dose and this was associated with reduced CYP2E1 immunolocalization in mitochondria [76]. The mechanism whereby repeated doses of coumarin induce lower mtCYP2E1 levels was not determined in this study.

### 4.3. NAFLD

**Figure 2:**
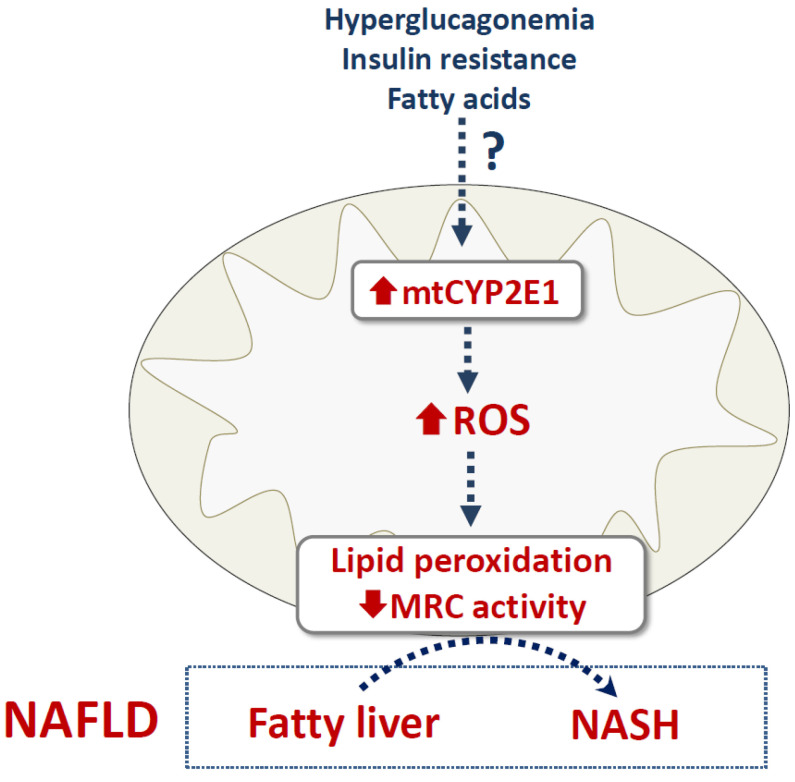
Hypothetical scheme regarding regulation and role of mitochondrial CYP2E1 in nonalcoholic fatty liver disease. In nonalcoholic fatty liver disease (NAFLD), different factors might favor CYP2E1 targeting to mitochondria including hyperglucagonemia, insulin resistance and some fatty acids. Higher levels of mitochondrial CYP2E1 (mtCYP2E1) might then secondarily cause reactive oxygen species (ROS) overproduction, which in turn triggers local lipid peroxidation and decreased activity of the mitochondrial respiratory chain (MRC). Of note, impaired MRC activity enhances ROS production, thus creating a vicious circle (not shown). In NAFLD, ROS and lipid peroxidation are deemed to play a key role in the transition of simple fatty liver to nonalcoholic steatohepatitis (NASH). Importantly, mtCYP2E1 involvement in NAFLD does not exclude the role of microsomal CYP2E1 in ROS overproduction, lipid peroxidation and mitochondrial dysfunction.

As mentioned above, there is growing evidence that CYP2E1 plays a role in the progression of obesity-associated fatty liver to NASH [2,3,18,37,38]. Investigations in wild-type and PPAR-α-null mice fed a standard or a high fat diet (HFD) showed that the highest level of hepatic mtCYP2E1 was found in Ppara-null HFD mice, which presented the highest NAFLD activity score (NAS) among all groups of animals [77]. Although these results suggest that increased mtCYP2E1 levels might favor the development of NASH, further investigations are required to reproduce these findings, clarify the specific role of mtCYP2E1 in NAFLD progression and eventually determine the mechanism of mtCYP2E1 induction. Of note, NAFLD seems to be commonly associated with hyperglucagonemia [78,79], which might contribute at least in part to the induction of hepatic mtCYP2E1 (Figure 2). It would also be important to determine whether insulin resistance can favor increased mtCYP2E1 levels. Although some findings suggest that insulin resistance might induce higher CYP2E1 expression and activity in NAFLD [3,32], further studies are required to determine whether this metabolic state specifically affects CYP2E1 targeting to mitochondria.

## 5. Conclusions and Future Perspectives

Hepatic CYP2E1 has long been known to be pivotal in the biotransformation and toxicity of several compounds such as ethanol, APAP and carbon tetrachloride, as previously mentioned. Although this toxicity is mostly secondary to the formation of reactive metabolites, CYP2E1 induction could also play a role by itself via ROS production from molecular oxygen. Alcohol abuse is a prototypical situation associated with CYP2E1 induction and CYP2E1-generated ROS but there is increasing evidence that other xenobiotics can also enhance CYP2E1 expression and activity in liver or other tissues. For instance, this has been shown for the industrial toxicants vinyl chloride and 1,4-dioxane [80,81] and for different pesticides including methoxychlor, phoxim, atrazine, maneb and paraquat [82,83,84,85,86]. A recent study also reported hepatic CYP2E1 induction in rats treated with the endocrine disruptor bisphenol A [87]. Finally, CYP2E1 can be induced by different pharmaceuticals such as isoniazid, phenobarbital, rifampicin and caderofloxacin [88,89,90,91]. Unfortunately, the aforementioned investigations did not determine whether these xenobiotics increased mtCYP2E1 expression and if so, whether mtCYP2E1 induction correlated with their toxicity. This is however a key issue, especially for xenobiotics able to induce liver steatosis [92,93] because CYP2E1-generated ROS are thought to promote the progression of simple steatosis to steatohepatitis, at least in part by inducing mitochondrial respiratory chain dysfunction and lipid peroxidation (Figure 2) [18,94].

Likewise, it will be important to determine whether hepatic mtCYP2E1 induction is a frequent feature of NAFLD. Indeed, CYP2E1 localization to mitochondria is expected to be detrimental on the respiratory chain [5], whose activity wanes during disease progression towards NASH [18,95,96]. If mtCYP2E1 induction is commonly found in NAFLD, further investigations will thus be required to determine the involved mechanism(s). In addition to hyperglucagonemia and insulin resistance, the role of some fatty acids should be considered (Figure 2). For instance, stearic acid alone or in association with oleic acid enhanced CYP2E1 activity in differentiated HepaRG cells [97,98]. Interestingly, although the activity of the respiratory chain complexes I, II and IV was normal in HepaRG cells incubated with stearic and oleic acids, such activity was significantly reduced when the steatotic cells were oriented towards a steatohepatitis-like state with a mixture of ethanol and benzo[a]pyrene [98]. Hence, these cellular models of NAFLD with CYP2E1 induction might prove useful to determine whether stearic acid and possibly other fatty acids could favor CYP2E1 targeting to mitochondria and the related mechanisms. A better understanding of the mechanisms leading to mtCYP2E1 localization in NAFLD might also help to find therapeutic strategies to curb the transition of fatty liver to NASH, cirrhosis and HCC. This is an urgent goal because it is currently estimated that NAFLD occurs in 25% to 30% of the adult population worldwide [99], while no specific drug for NAFLD has yet been approved by regulatory agencies [100].
